# Diversity and phenotypic analyses of salt- and heat-tolerant wild bean *Phaseolus filiformis* rhizobia native of a sand beach in Baja California and description of *Ensifer aridi* sp. nov.

**DOI:** 10.1007/s00203-019-01744-7

**Published:** 2019-10-28

**Authors:** Guadalupe Rocha, Antoine Le Queré, Arturo Medina, Alma Cuéllar, José-Luis Contreras, Ricardo Carreño, Rocío Bustillos, Jesús Muñoz-Rojas, María del Carmen Villegas, Clémence Chaintreuil, Bernard Dreyfus, José-Antonio Munive

**Affiliations:** 1 Centro de Investigaciones en Ciencias Microbiológicas, Instituto de Ciencias, Benemérita Universidad Autónoma de Puebla, Av. San Claudio S/N, CP-72570 Puebla, Mexico; 2 IRD/CIRAD/UM2/Supagro-UR 040 Laboratoire des Symbioses Tropicales et Méditerranéennes, 34398 Montpellier, France; 3 Facultad de Arquitectura, Benemérita Universidad Autónoma de Puebla, Av. San Claudio S/N, CP-72570 Puebla, Mexico; 4 Helyx Affaires SC, Rumania 923-2. Col. Portales-Sur. Alcaldía Benito Juárez, CP-03300 Cd. de México, Mexico

**Keywords:** Legume–rhizobium symbiosis, *Ensifer*, Wild bean, Salt tolerance

## Abstract

In northern Mexico, aridity, salinity and high temperatures limit areas that can be cultivated. To investigate the nature of nitrogen-fixing symbionts of *Phaseolus filiformis*, an adapted wild bean species native to this region, their phylogenies were inferred by MLSA. Most rhizobia recovered belong to the proposed new species *Ensifer aridi*. Phylogenetic analyses of *nodC* and *nifH* show that Mexican isolates carry symbiotic genes acquired through horizontal gene transfer that are divergent from those previously characterized among bean symbionts. These strains are salt tolerant, able to grow in alkaline conditions, high temperatures, and capable of utilizing a wide range of carbohydrates and organic acids as carbon sources for growth. This study improves the knowledge on diversity, geographic distribution and evolution of bean-nodulating rhizobia in Mexico and further enlarges the spectrum of microsymbiont with which *Phaseolus* species can interact with, including cultivated bean varieties, notably under stressed environments. Here, the species *Ensifer aridi* sp. nov. is proposed as strain type of the Moroccan isolate LMR001^T^ (= LMG 31426^T^; = HAMBI 3707^T^) recovered from desert sand dune.

**Electronic supplementary material:**

The online version of this article (10.1007/s00203-019-01744-7) contains supplementary material, which is available to authorized users.

## Introduction

The Leguminosae (Fabaceae) is the third largest family of flowering plants with approximately 730 genera and over 19,400 species (Mabberly 1997; Lewis et al. 2005). Grain legumes such as soybeans, beans, peas, or peanuts are agriculturally and economically important. Their particularly high protein content represents an ecological alternative to cattle farming. Furthermore, their capacity to develop a natural mutualistic interaction with symbiotic nitrogen-fixing bacteria (referred as rhizobia) can reduce nitrogen fertilizer use which requires, however, the presence of compatible, competitive and efficient microbial partners.

Common bean (*Phaseolus vulgaris* L.) represents the most important source of proteins for low-income populations in Latin America and Africa (Ribeiro et al. 2009). The genus *Phaseolus* comprises around 50 species, all indigenous to the Americas. Among these, *P. lunatus*, *P. vulgaris*, *P. polyanthus*, *P. coccineus* and *P. acutifolius* were domesticated by pre-Hispanic civilizations and are now widely cultivated for human consumption. Given the potential interest in promoting natural symbioses for plant growth improvement and adaptation to a changing environment, there is interest in investigating the rhizobial partners that can develop the symbiosis with wild bean adapted to stressed environments, compare their diversity and estimate their symbiosis capacities with those of cultivated beans’ symbionts. *Phaseolus filiformis* (also named slimjim bean, slender-stem bean, or Wright’s phaseolus), is a wild bean species native to the southwestern United States and northwestern Mexico, a region subjected to frequent droughts and salt stresses (Shreve and Wiggins 1964; Hickman 1993; Roskov et al. 2013).

Most of the described rhizobial species belong to the subclass Alphaproteobacteria (Lindström and Mousavi 2010; Lindström 2015), but there are also a few representatives within the subclass Betaproteobacteria also called beta-rhizobia (Moulin et al. 2001; Chen et al. 2003). Most reported phylogenies of rhizobia nodulating *Phaseolus* species have placed them in the genera *Rhizobium* and *Bradyrhizobium* (Servín-Garcidueñas et al. 2014). Even though most legumes reported in the tribe Phaseoleae are nodulated by *Bradyrhizobium* strains (Martinez-Romero 2009), *P. vulgaris* and closed species are mainly nodulated by *Rhizobium* strains. However, studies have shown that strains belonging to the genus *Ensifer* (*Sinorhizobium*) (*E. fredii*, *E. meliloti* and *E. americanum*) (Herrera-Cervera et al. 1999; Mnasri et al. 2007; Zurdo-Piñeiro et al. 2009; Verástegui-Valdés et al. 2014) can also develop an effective symbiosis with this crop and more recently the beta-rhizobium *Paraburkholderia nodosa* was found as the main symbiotic partner in the Brazilian Cerrado region (Dall’Agnol et al. 2016).

Due to the need for easy, robust and reliable techniques for the identification and classification of bacterial isolates at the species level, phylogenetic analyses using several conserved and single copy protein-encoding genes (housekeeping genes), with a higher level of sequence divergence than rRNA genes are now encouraged. The ad hoc committee for the redefinition of bacterial species concept has recommended the use of several genes to perform such classification (Stackebrandt et al. 2002). More recently, multilocus sequence analysis (MLSA) which uses a concatenated sequence of multiple markers to infer evolutionary relationships among bacterial isolates was shown as a reliable method to classify species in several genera including *Ensifer* (Martens et al. 2007, 2008) or *Bradyrhizobium* (Vinuesa et al. 2008).

The aim of the present work was to isolate and characterize rhizobia nodulating the wild bean species *P. filiformis* and test their capacities to develop the symbiosis with cultivated beans. Isolated strains were genotyped by restriction patterns produced by ARDRA and REP-PCR. To clarify their taxonomic position within the phylogeny of rhizobia, several taxonomic markers (16S rDNA, *atpD*, *dnaK*, *rpoB*, *glnA*, *gyrB*, *recA*, *thrC*, and *glnII*) were partially sequenced and analyzed. In addition, two symbiotic genes (*nifH* and *nodC*), that reflect the symbiotic properties of rhizobia (Haukka et al. 1998; Laguerre et al. 2001), were studied. This collection was also phenotypically characterized for their capacity to nodulate various hosts including several cultivated bean varieties, carbon source utilization, and tolerance to alkalinity, salinity, and high-temperature conditions.

## Materials and methods

### Origin of the strains and culture conditions

*Phaseolus filiformis* plants were sampled in the desertic region of Baja California, Mexico (22°55′51.5″N, 109°48′55.3″W), at 9.5 m above sea level on the Santa Maria Bay, Guadalupe Beach. The plants were grown on sandy soil presenting an electrical conductivity of 6.276 ds m^−1^ (saline soil), an alkaline pH (H_2_O) of 7.82 and presented a low cation exchange capacity of 3.16 meq^+^/100 g. The soil was poor, containing 0.07% of nitrogen, while organic matter was not detectable. All the plants sampled were nodulated. Rhizobial strains were isolated from nodules using the standard procedures described by Vincent (1970), on yeast mannitol agar (YMA) medium (Bergersen 1961). Strains were grown on YMA at 28 °C and kept in 20% (v/v) glycerol at − 80 °C. For comparison, we used *E. saheli* LMG8310 (ORS611), kindly provided by Esperanza Martínez-Romero (Center for Genome Sciences, UNAM, México).

### Nodulation tests

All strains were evaluated for their ability to induce nodules on different host legume species. Plants tested included their natural host (*P. filiformis*), five different *Phaseolus vulgaris* varieties (Negro jamapa, peruano, pinto, vaquita and alubia) and five different legume genera (*Mimosa pudica, Erythrina americana, Medicago sativa, Cicer arietinum* and *Lupinus gredensis*). *Phaseolus* seeds were surface sterilized as described previously (Beynon et al. 1980). *Erythrina* seeds were rinsed in water, scarified mechanically using sandpapers and sterilized by immersion in concentrated sulfuric acid for 10 min, rinsed with sterile water, and left in water overnight. *Mimosa* seeds were rinsed in water, sterilized by immersion in concentrated sulfuric acid for 10 min, rinsed with sterile water, and left in water overnight. *Medicago* and *Lupinus* seeds were submerged in 70% alcohol for 10 min, immersed in 25% sodium hypochlorite solution for 15 min and rinsed with sterile water. *Cicer* seeds followed the latter protocol, but they were soaked in a boiling water bath for 50 s prior to treatment exposure (modified from Somasegaran and Hoben 1994).

Seeds were placed onto water agar (0.75% w/v) in Petri dishes until germination. Germinated seedlings were transferred to agar slants (20 ml of agar in 50 ml tube) of Jensen’s N-free medium (Somasegaran and Hoben 1994). Plants were incubated at 20 °C with a 16-h light, 8-h dark cycle (Knight et al. 1986). A single colony of each isolate was picked from YMA plates and grown aerobically in yeast extract mannitol broth (YM) on an orbital shaker (180 rpm) at 28 °C. Each seedling was inoculated with 1 ml bacterial culture at exponential growth phase (Somasegaran and Hoben 1994). For each plant species tested, controls consisted of non-inoculated seedlings, either supplied with mineral nitrogen (as 0.1% KNO_3_ in nutrient solution) or grown without nitrogen. Roots of plants were observed for root nodule formation 3–4 weeks after inoculation.

### PCR amplification and sequencing of housekeeping genes for MLSA, ribosomal and symbiotic genes

Total DNA was extracted using the Wizard Genomic DNA Purification Kit (Promega Corporation, USA), according to the manufacturer´s protocol. PCR was performed in a 2400 GeneAmp PCR Systems^®^ Perkin Elmer thermocycler. Internal fragments of the housekeeping genes, *atpD* (ATP synthase F1, beta subunit) and *glnII* (glutamine synthetase II) (Vinuesa et al. 2005); *rpoB* (RNA polymerase, beta subunit) and *gyrB* (DNA gyrase B subunit) (Martens et al. 2008); *recA* (recombinase A) (Gaunt et al. 2001), *dnaK* (heat shock protein HSP70) (Stepkowski et al. 2003); *glnA* (glutamine synthetase type I) and *thrC* (threonine synthase) (Martens et al. 2007), as well as the 16S rDNA gene, and the symbiosis-related genes *nodC* (*N*-acetylglucosaminyltransferase) and *nifH* (dinitrogenase reductase) (Laguerre et al. 2001), were amplified using the conditions described in Table 1.

**Table 1:** Primers and PCR cycling conditions used in this work

Primer	Sequence (5′–3′)	PCR cycling conditions	References
REPIR-l REP2-1	IIIICGICGICATCIGGC ICGICTTTATCIGGCTAC	7′ 95 °C, × 35 (1′ 94 °C, 1′ 40 °C, 8′ 65 °C), 16′ 65 °C, ∞ 20 °C	Versalovic et al. (1994)
UN27F UN1392R	TAGAGTTTGATCCTGGCTCAG CAGGGGCGGTGTGTACA	3′ 95 °C, × 26 (30″ 94 °C, 1′ 57 °C, 1′10″ 72 °C), 10′ 72 °C, ∞ 20 °C	Biodiversa Inc., México
atpD255F atpD782R	GCTSGGCCGCATCMTSAACGTC GCCGACACTTCMGAACCNGCCTG	3′30″ 95 °C, × 30 (1′ 93.5 °C, 40″ 55 °C, 1′ 72 °C), 5′ 72 °C, ∞ 15 °C	Vinuesa et al. (2005)
glnII-12F glnII-689R	YAAGCTCGAGTACATYTGGCT TGCATGCCSGAGCCGTTCCA	3′30″ 95 °C, × 30 (1′ 93.5 °C, 1′ 58 °C, 1′ 72 °C), 5′ 72 °C, ∞ 15 °C	Vinuesa et al, 2005
TSdnaK2 TSdnaK4	GTACATGGCCTCGCCGAGCTTCA GGCAAGGAGCCGCAYAAGG	5′ 94 °C, × 35 (30″ 94 °C, 1′ 60 °C, 45″ 72 °C), 7′ 72 °C, ∞ 20 °C	Stepkowsky et al. (2003)
glnA144F glnA1142R	GTCATGTTCGACGGYTCYTCG TGGAKCTTGTTCTTGATGCCG	3′30″ 95 °C, × 30 (1′ 93.5 °C, 1′ 61 °C, 1′ 72 °C), 5′ 72 °C, ∞ 20 °C	Martens et al. (2007)
gyrB343F gyrB1043R	TTCGACCAGAAYTCCTAYAAGG AGCTTGTCCTTSGTCTGCG	5′ 96 °C, × 35 (30″ 94 °C, 30″ 59 °C, 1′ 72 °C), 3′ 72 °C, ∞ 20 °C	Martens et al. (2008)
recA12^a^ recA555	GTAGAGGAYAAATCGGTGGA CGRATCTGGTTGATGAAGATCACCAT	5′ 94 °C, × 35 (30″ 94 °C, 1′ 50 °C, 45″ 72 °C), 7′ 72 °C, ∞ 20 °C	Gaunt et al. (2001)
rpoB83F rpoB1061R	CCTSATCGAGGTTCACAGAAGGC AGCGTGTTGCGGATATAGGCG	5′ 96 °C, × 35 (30″ 94 °C, 30″ 59 °C, 1′ 72 °C), 3′ 72 °C, ∞ 20 °C	Martens et al. (2008)
thrC577F thrC1231R	GGCAMKTTCGACGAYTGCCAG GGRAATTTDGCCGGRTGSGC	3′30″ 95 °C, × 30 (1′ 93.5 °C, 1′ 55 °C, 1′ 72 °C), 5′ 72 °C, ∞ 20 °C	Martens et al. (2007)
nodCF nodCI	AYGTHGTYGAYGACGGTTC CYGGACAGCCANTCKCTATTG	5′ 95 °C, × 35 (30″ 94 °C, 30″ 56 °C, 1′ 72 °C), 7′ 72 °C, ∞ 20 °C	Laguerre et al. (2001)
nifHF nifHI	TACGGNAARGGSGGNATCGGCAA AGCATGTCYYCSAGYTCNTCCA	5′ 95 °C, × 35 (30″ 94 °C, 30″ 60 °C, 45″ 72 °C), 7′ 72 °C, ∞ 20 °C	Laguerre et al. (2001)

^a^Modified from Gaunt et al. (2001)

PCR products and their concentration were verified by electrophoresis on a 1% agarose gel stained with ethidium bromide. A molecular size marker (GeneRuler 1 Kb DNA Ladder) was included to estimate the length of the amplification products. The amplified products were purified to remove salts, polymerase and excess primers and nucleotides, using a Qiaquick PCR purification kit (Qiagen) according to the manufacturer’s instructions. Concentration of purified products was measured in a spectrophotometer NanoDrop^®^ ND-1000 (NanoDrop Technologies, Wilmington, DE). Sequencing was performed by the Genoscreen company service (Applied Biosystems 3730XL DNA sequencer). Consensus sequences were obtained using the AutoAssembler software (Applied Biosystems).

### Sequence alignment and phylogenetic analyses

Sequences were compared with those of the selected strains including type material available from Genbank database at the time of writing. The GenBank accession numbers are included in supplementary materials (Supp. Table S1 and Supp. Table S2). Concatenated sequences were generated using DNAsp v5 (Librado and Rozas 2009). Nucleotide sequence alignments were made using CLUSTAL_X (Thompson et al. 1997), and corrected manually using GeneDoc (Nicholas et al. 1997). Maximum-likelihood (ML) analyses were performed with MEGA version 7.0 (Kumar et al. 2016; Tamura et al. 2011). ML analyses were performed using the models with the lowest Bayesian information criterion score. All analyses were performed with at least 500 bootstrap replications.

### ARDRA and REP-PCR genomic fingerprinting

PCR-based locus-specific RFLP ribotyping can be successfully applied for the differentiation of bacterial strains that display a low degree of heterogeneity within the rRNA operons (Olive and Bean 1991). Amplified 16S rDNA was digested with the restriction enzymes *Alu*I*, Cfo*I, *Hinf*I and *Nhe*I purchased at Roche Diagnostics GmbH (Germany). The digested DNA was separated in 2.5% (w/v) agarose gel, for 3 h at a constant voltage of 70 V cm^−1^ at 4 °C, and further stained with ethidium bromide. REP-PCR generates DNA fingerprints that allow the discrimination of bacterial strains (Versalovic et al. 1994). The REP-PCR amplifications were performed using the conditions described in Table 1. The REP-PCR profiles were visualized under ultraviolet light after staining with ethidium bromide. The resulting fingerprints were analyzed by the BioNumerics V4.0 software package (Applied Maths, Ghent, Belgium). The similarity among digitized profiles was calculated using the Pearson correlation, and an average linkage (UPGMA) dendrogram was derived from the profiles.

### Effect of temperature, salt concentration and pH on bacterial growth

To estimate the tolerance of isolated rhizobia to salt, *Ensifer* strains were first pre-cultivated at 28 °C for 24 h in 5 ml of YM broth (pH 6.8) with shaking (180 rpm). This volume was then used to inoculate 50 ml of YM media to reach a target initial titer of 8 × 10^8^ CFU/ml. Bacterial tolerance was estimated by inoculating modified YMA medium containing either 0.85 mM, 10 mM, 50 mM, 100 mM, 150 mM, 200 mM, 300 mM, 400 mM, 500 mM, 600 mM, 800 mM or 1000 mM NaCl. Plates were incubated at 28 °C for 7 days, and colony-forming units per milliliter (CFU/ml) were calculated for each sample by massive stamping drop plate technique (Corral-Lugo et al. 2012). For the analysis of temperature and pH effects on bacterial growth, the strains were pre-cultivated as previously described but incubated at different temperatures from 28 °C, 32 °C, 37 °C up to 40 °C, or at different pHs (5.3, 6.8, 8.3 and 9.5). Bacterial growth was estimated as previously described (Corral-Lugo et al. 2012).

### Determination of carbon assimilation profiles

Oxidation of 48 carbon sources was tested with the API 50 CH systems (API strips, Biomérieux, France) using overnight YM broth cultures grown at 28 °C according to the manufacturer’s protocol. The results were recorded after 24 h and 48 h of incubation at 28 °C. Each strain was tested in API 50CH on two separate occasions and showed good reproducibility.

### Genome-based species delineation

The average nucleotide identity (ANI) (Goris et al. 2007) and digital DNA–DNA hybridization (dDDH) estimates (Meier-Kolthoff et al. 2013) were used to determine the molecular delineation of *Ensifer aridi* strains from neighboring species. The accession numbers of all genome sequences used here are reported in Supplementary table S3. These included the genome sequences of strains LEM451 and LEM457 representing the majority of strains recovered from *P. filiformis* as well as LMR001^T^ and LMR013 recovered from the Merzouga desert in Morocco and TP6 and TW10 isolated from the Indian Thar desert. These genomic sequences were compared to available genome sequences from all *Ensifer* type strains available at the time of writing including that of *E. saheli* LMG 7837^T^, the closest relative of *Ensifer aridi* (Supplementary table S3). For the ANI calculation, alignment options used were 70% minimal identity over more than 700 bp in fragment windows of 1000 bp with a 200 bp step size on reciprocal best hits (two-way ANI). For the dDDH estimation, we used the Genome to Genome Distance Calculator 2.1 (GGDC) with the BLAST + alignment method which uses the generalized linear model with the recommended settings (Meier-Kolthoff et al. 2013). The TYGS method using recommended settings was used to show species delineation and for phylogenomic tree construction (Meier-Kolthoff and Göker 2019).

## Results

### Isolation of rhizobia and nodulation tests

*Phaseolus filiformis* was the primary natural legume plant present on the sampled site. Ten rhizobial strains were isolated from nodules of *P. filiformis* directly growing in the sand and tested for nodulation on eleven different host legumes. Our tests did not reveal any variation of their respective nodulation phenotype. All these strains were capable of nodulating their original host, *P. filiformis*, as well as all the bean varieties tested. However, using the culture conditions used here, they were not capable of nodulating the other legumes tested (*M. pudica, E. americana, M. sativa, C. arietinum* nor *L. gredensis*).

### Genomic fingerprinting

The PCR–RFLP of the 16S rDNA genes and a REP-PCR genomic fingerprinting of rhizobia recovered were carried out. Strains were categorized in ten genotypes by combination of the restriction patterns from the four enzymes and from REP-PCR fingerprinting patterns (data not shown). Given the high diversity obtained, all isolated strains were further analyzed.

### Phylogenetic analyses of *P. filiformis* symbiotic isolates

**Fig. 1:**
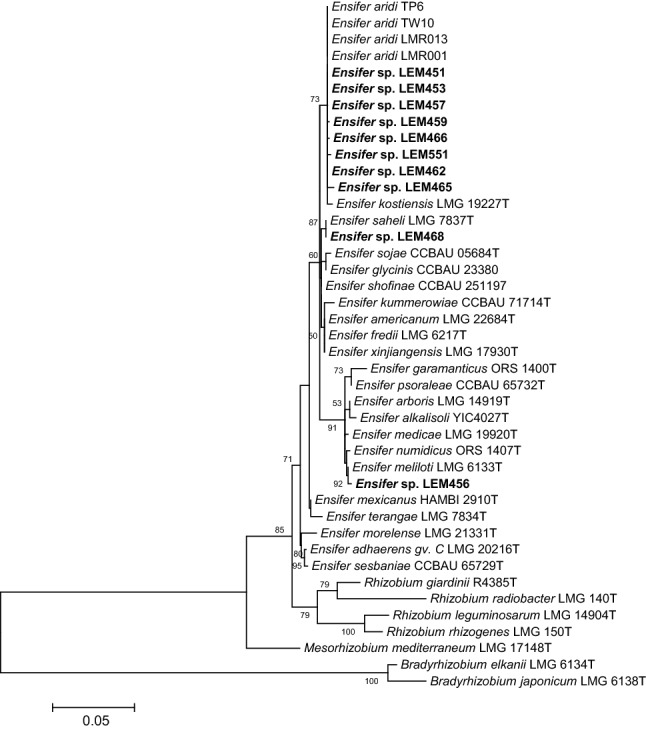
Maximum-likelihood tree based on partial 16S rDNA gene sequences showing the evolutionary relationships between *Ensifer* strains isolated from *Phaseolus filiformis* nodules in Mexico (bold) and reference strains. The analysis was based on 1229 nucleotides alignment. The significance of each branch is indicated by the bootstrap value calculated for 500 replicates (only values higher than 50% are indicated). Scale bar indicates 5% substitution per site. Accession numbers of sequences used are reported in supplementary Table S1

The maximum-likelihood tree based on nearly full 16S rDNA gene sequences (Fig. 1) showed that the *P. filiformis* isolates belonged to the *Ensifer* genus and grouped into three different clusters. The majority of the partial 16S rDNA sequences (8/10) showed high sequence similarities to those of *Ensifer* strains recovered from Asian or African deserts recently described, and for which the name “*Ensifer aridi*” was proposed based on genomic data which included the two *P. filiformis* rhizobial strains LEM451 and LEM457 (Le Queré et al. 2017). The 16S rDNA sequences of the strains LEM451, LEM453, LEM457 and LEM462 were 100% identical over 1227 bp aligned sequences to that of African and Asian “*Ensifer aridi”* isolates; LEM459 and LEM466 presented a single substitution, while LEM551 and LEM465 presented 2 and 4 substitutions, respectively. The partial 16S rDNA sequence of one isolate (LEM468) was identical to that of *Ensifer saheli* LMG 7837^T^ that is closely related to the “*Ensifer aridi*” clade with which it shares 1220 bases out of the 1227 sequenced. Finally, LEM456 was closely related to *E. meliloti*; differing only 2 nucleotides along the 1227 aligned sequences (99.8% identity).

**Fig. 2:**
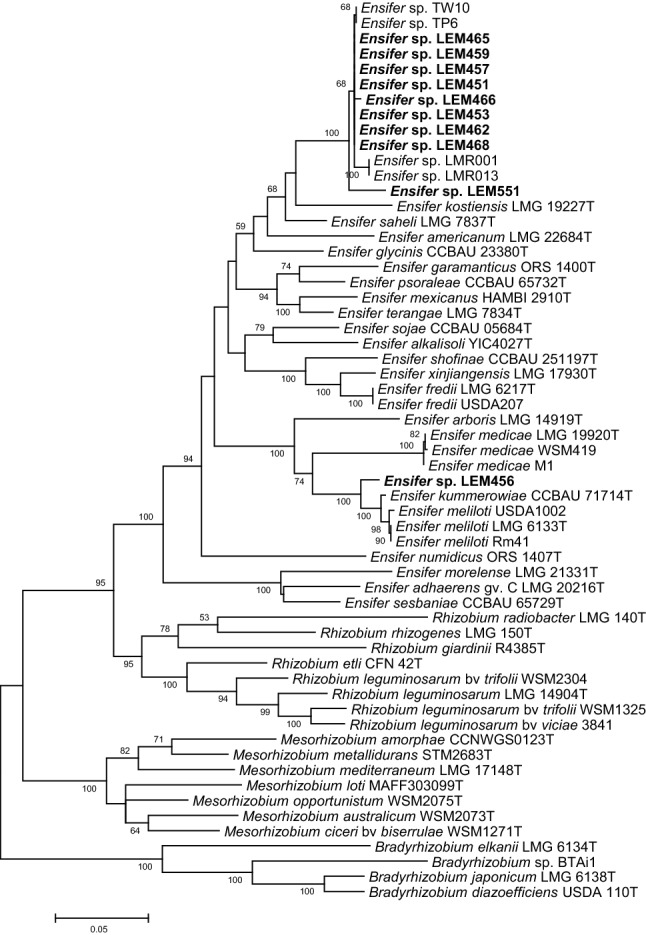
Maximum-likelihood tree based on concatenated *atpD*–*recAglnII* partial gene sequences showing the evolutionary relationships between *Ensifer* strains isolated from *Phaseolus filiformis* nodules in Mexico (bold) and reference strains. The analysis was based on 1245 nucleotides alignment. The significance of each branch is indicated by the bootstrap value calculated for 500 replicates (only values higher than 50% are indicated). Scale bar indicates 5% substitution per site. Accession numbers of sequences used are reported in supplementary Table S1

To better resolve the position of the strains among *Ensifer* species, we then constructed a phylogenetic tree upon concatenation of three housekeeping genes (*atpD*, *recA* and *glnII*) that are available for most of *Ensifer* species (Fig. 2). The tree obtained presented several differences with that obtained based on the 16S rDNA phylogeny. In particular, the phylogenetic proximity between strain LEM468 and *E. saheli*, and that obtained between the rest of Mexican strains (except LEM456) and *E. kostiense* were not confirmed. The strain LEM468, that showed partial 16S rDNA sequence identical to *Ensifer saheli* LMG 7837^T^, clustered together with the strains of the “*Ensifer aridi”* clade. Furthermore, in contrast to the 16S rDNA sequence-based phylogeny, this large group of *Ensifer* strains presented a higher sequence similarity with *E. saheli* than with *E. kostiense,* even though the latter species clustered together within a clade that encompassed *E. aridi* and *E. saheli* strains which was supported by a bootstrap of 68%. Finally, the strain LEM456 was separated from the remaining Mexican isolates and was related to the species *E. kummerowiae* and *E. meliloti*, which is in good agreement with the 16S rDNA phylogeny.

Individual analyses of the eight housekeeping genes partially sequenced showed that the *recA* gene presented the highest similarity with 100% identity between sequences of the nine Mexican strains clustering with “*Ensifer aridi”*. The partial *rpoB* and *atpD* gene sequences were identical among these nine strains except for LEM466 which presented one and four substitutions, respectively. The *gyrB* sequences presented from 99.6 to 100% sequence similarities and the *thrC* alignment presented from 99 to 100% sequence similarities among this group of nine *P. filiformis* isolates. The partial gene sequences of *glnII* were identical for this group of strains except for LEM551 which showed 94.9% sequence similarity. In contrast to these six housekeeping genes, the alignment of *dnaK* partial sequences presented more divergent sequences for strains LEM466 and LEM551 which presented, respectively, 91.9 and 86.3% sequence similarities with the remaining sequences from the strains that clustered within the *E. aridi* clade. The *glnA* partial sequence alignment showed 90.6–100% sequence similarities between these nine strains. The sequence similarities between LEM456 and *E. meliloti* strains were also high. Indeed, when comparing these eight housekeeping gene sequences between LEM456 and six *Ensifer meliloti* strains, the *recA* gene presented similarity ranging from 97.3 to 100%. For the remaining markers, *dnaK*, *rpoB*, *atpD*, *gyrB*, *thrC*, *glnA* and *glnII*, similarities ranged, respectively, from 97.5 to 99.5%, 97.4 to 99.1%, 96.8 to 98.4%, 96.9 to 99.8%, 97.6 to 99.8%, 96.8 to 99.7% and 97.7 to 100%.

**Fig. 3:**
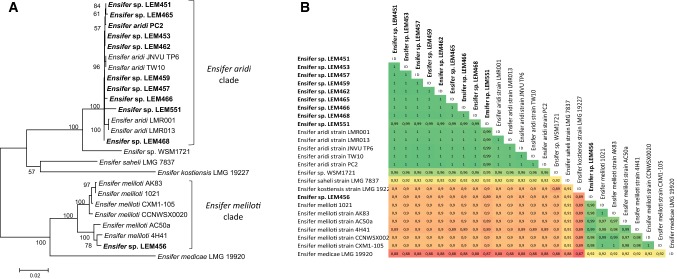
Analysis of concatenated *atpD*–*glnII*–*recA*–*gyrB*–*rpoB*–*thrC* partial gene sequences between *Ensifer* strains isolated from *Phaseolus filiformis* nodules in Mexico (bold) and closely related strains and species. **a** Maximum-likelihood tree showing the evolutionary relationships. The analysis was based on 3213 nucleotides alignment. The significance of each branch is indicated by the bootstrap value calculated for 500 replicates (only values higher than 50% are indicated). Scale bar indicates 2% substitution per site. **b** Matrix showing pairwise percentage of similarities between compared strains and species. The percentage of similarities between sequences from two strains is highlighted in red (most divergent) to dark green (most conserved). Accession numbers of sequences used are reported in supplementary Table S1

Analysis of the alignment obtained from concatenated sequences of the six housekeeping genes *recA*, *atpD*, *gyrB*, *rpoB*, *glnII* and *thrC* from the ten Mexican isolates with strains from the closest *Ensifer* species identified were performed (Fig. 3). This confirmed that nine of the strains are closely related to the species “*Ensifer aridi”* forming a branch with strong bootstrap support (100%) with which they shared 98.9–99.9% identity over the 3213 bp concatenated sequences (Fig. 3). Using this alignment, the strain LEM456 clustered clearly (bootstrap of 100%) within the clade that included the six *E. meliloti* strains and with which it shared from 97.5 to 99.3% sequence similarities.

### Analysis of symbiotic *nodC* and *nifH* genes

**Fig. 4:**
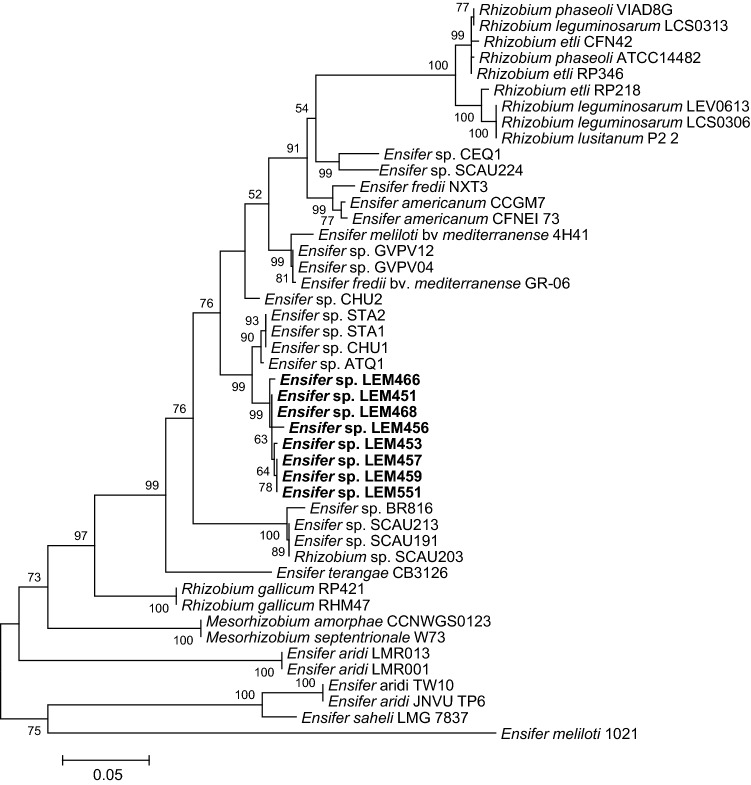
Maximum-likelihood tree based on *nodC* partial gene sequences showing the evolutionary relationships between *Ensifer* strains isolated from *Phaseolus filiformis* nodules in Mexico (bold) and selected strains. The analysis was based on 722 nucleotides alignment. The significance of each branch is indicated by the bootstrap value calculated for 500 replicates (only values higher than 50% are indicated). Scale bar indicates 5% substitution per site. Accession numbers of sequences used are reported in supplementary Table S2

**Fig. 5:**
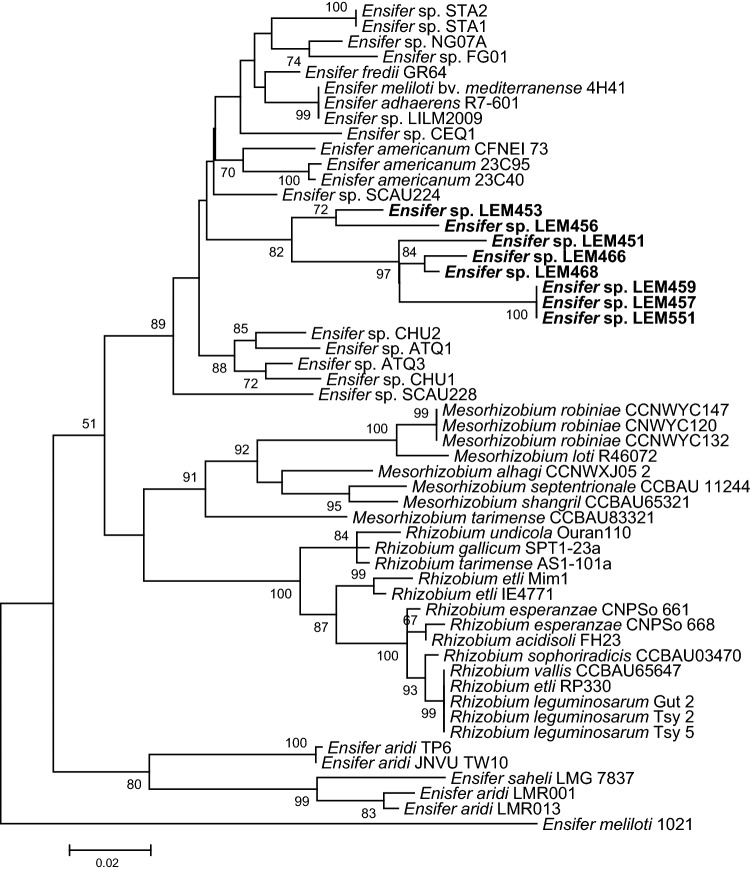
Maximum-likelihood tree based on *nifH* partial gene sequences showing the evolutionary relationships between *Ensifer* strains isolated from *Phaseolus filiformis* nodules in Mexico (bold) and selected strains. The analysis was based on 652 nucleotides alignment. The significance of each branch is indicated by the bootstrap value calculated for 500 replicates (only values higher than 50% are indicated). Scale bar indicates 5% substitution per site. Accession numbers of sequences used are reported in supplementary Table S2

Symbiotic genes reflect the bacterial symbiotic properties of rhizobia (Haukka et al. 1998; Laguerre et al. 2001). To explore the symbiotic diversity of *P. filiformis* isolates, two symbiotic genes (*nodC* and *nifH*) were partially sequenced for eight isolates and their phylogenies compared to those obtained using housekeeping genes. Based on differences along a 722 pb DNA fragment of *nodC*, all rhizobial isolates compared grouped together in a single clade related to *nodC* sequences from *Ensifer* strains isolated from *Acacia macracantha* (*Vachellia macracantha*) nodules in Peru (Fig. 4, Supp. Table S2). The genetic diversity of a 652 pb DNA fragment corresponding to the partial sequences of the nitrogen fixation gene *nifH* among the Mexican *Ensifer* strains reported here showed a greater sequence divergence. However, they grouped within a large clade of *Ensifer* strains belonging to *E. americanum*, *E. meliloti, E. fredii* or yet undefined *Ensifer s*pecies recovered from *Phaseolus vulgaris* in Europe, Africa, Asia or America, as well as those isolated from *Acacia macracantha* nodules in Peru or from *Leucaena leucocephala* in arid-hot river valley area in Panxi, Sichuan, China (Fig. 5, Supp. Table S2).

### Effect of salt stress on bacterial growth

Capable of growing directly on the beach, at sea level, we supposed that the wild bean species *P. filiformis* and its symbiotic partners were able to tolerate high salt concentrations. Here, the tolerance to saline stress for the isolated strains was evaluated using *E. saheli* LMG 8310 (the closest related species) as control. We confirmed that all *Ensifer* strains tested were salt tolerant (Table 2). All *Ensifer* strains tested presented good growth up to 300 mM NaCl. Six strains LEM451, LEM456, LEM457, LEM459, LEM465, LEM466 and *E. saheli* LMG 8310 can grow at 400 mM NaCl, but strains LEM456, LEM457, LEM459 and LEM465 showed weak growth at this concentration. Strains LEM457 and LEM459 did not stand higher NaCl concentrations, while strains LEM456 and LEM 465 showed slight growth at 500 mM NaCl. The growth of *Ensifer* strain LEM466 was not affected by NaCl concentrations up to 600 mM but did not tolerate higher concentrations. Finally, strain LEM451 was the most tolerant to saline stress, showing weak growth up to 1000 mM NaCl concentration in the medium tested*. E. saheli* strain LMG 8310 showed weak growth at 500 mM and 600 mM NaCl concentrations above which growth was impaired.

**Table 2:** Effect of temperature, salt concentration and pH of medium on bacterial growth of *Ensifer* strains isolated from *P. filiformis* in Mexico

Strains	Bacterial growth in YMA at different temperature conditions (°C)				Bacterial growth in YMA with NaCl at different concentrations (mM)												Growth in YMA medium at different pH conditions			
	28	32	37	40	0.085	10	50	100	150	200	300	400	500	600	800	1000	5.3	6.8	8.3	9.5
Mexican strains																				
LEM451	++	++	++	++	++	++	++	++	++	++	++	+	+	+	+	W	W	++	++	++
LEM453	++	++	++	++	++	++	++	++	++	++	+	−	−	−	−	−	W	++	++	++
LEM551	++	++	++	++	++	++	++	++	++	++	+	−	−	−	−	−	+	++	++	+
LEM456	++	++	++	++	+	++	++	++	++	+	+	W	W	−	−	−	+	+	++	++
LEM457	+	++	++	+	+	+	+	+	+	+	+	W	−	−	−	−	−	++	++	++
LEM459	+	+	+	++	++	++	+	+	+	+	+	W	−	−	−	−	−	++	++	++
LEM462	++	++	++	++	++	++	++	++	++	+	+	−	−	−	−	−	+	++	++	++
LEM465	+	+	+	+	+	++	+	+	+	+	+	W	W	−	−	−	−	+	+	+
LEM466	+	+	+	+	+	+	+	+	+	+	+	+	+	+	−	−	−	+	+	+
LEM468	++	++	++	+	+	+	+	+	+	+	+	−	−	−	−		W	+	++	++
*E. saheli* LMG8310	+	++	++	++	++	++	++	++	++	++	++	+	W	W	−	−	+	++	++	++

++ Vigorous growth

+ Good growth

W Weak growth

− No growth

### Effect of temperature and pH on bacterial growth

The newly isolated strains were also characterized phenotypically for their tolerance to high temperatures and alkalinity parameters that are common in desert environments. All *Ensifer* strains were tolerant to high temperatures (Table 2), being unaffected at 28 °C, 32 °C, 37 °C and 40 °C. All strains isolated from *P. filiformis* and *E. saheli* were high-temperature tolerant.

In relation to the effect of pH on bacterial growth, and using *E. saheli* LMG 8310 as control, we confirmed that all strains were tolerant to alkaline conditions as they could grow at a pH of 9.5; nevertheless, so we found that most strains are sensitive to acidic pH (Table 2). The growth of *Ensifer* strains LEM551, LEM462, LEM456 and *E. saheli* LMG 8310 was unaffected by acidic conditions (pH 5.3). The same pH slightly affected the growth of strains LEM451, LEM453 and LEM468, meanwhile *Ensifer* strains LEM457, LEM459, LEM465 and LEM466 did not stand such acid pH.

### Determination of carbon utilization profiles

Ten strains isolated in the present report were characterized for their capacities to utilize a wide range of carbohydrates on API CH50 strips and compared to those of various strains of *Ensifer saheli*, *E. meliloti*, *E. fredii* and *E. terangae* including type strains (De Lajudie et al. 1994) or to Biolog GN2 phenotypic microplates obtained from *E. aridi* strains isolated from Indian Thar desert or the Moroccan Merzouga desert (Le Queré et al. 2017) (Supp. Table S4). Variability in carbon source utilization among *Ensifer* isolates and species was observed. These strains utilize most carbon sources tested (48 different carbon sources), but the patterns of carbon sources utilization were very diverse among the strains isolated from *P. filiformis*. All strains studied here were capable of utilizing d-mannitol, l-arabinose, ribose, methyl-βd-xylanopyranoside, d-maltose, d-lyxose and potassium-5-ketogluconate; while they were not capable of metabolizing glycogen. Even strain LEM456, the most divergent strain by MLSA, showed a similar carbon utilization profile. In contrast, most carbon sources tested were not utilized by strain LEM551 which was only capable of using these eight common carbon sources, as well as inositol and salicin that were also metabolized by most Mexican strains. These results indicate metabolic versatility of the wild bean rhizobia presented here. Finally, all the eight Mexican *Ensifer aridi* strains (LEM451, LEM453, LEM457, LEM459, LEM462, LEM465, LEM466 and LEM468) as well as the two Moroccan strains (LMR001, LMR013) and the two Indian strains (TP6 and TW10) were capable of utilizing glycerol, l-arabinose, d-fructose, l-rhamnose, inositol, d-mannitol, d-maltose, d-sucrose, d-trehalose, d-raffinose, d-turanose and d-arabitol. It should be noted that all these C sources were also metabolized by the strains of *Ensifer saheli*, *E. terangae*, *E. meliloti* and *E. fredii* tested by De Lajudie et al. (1994).

### Genome-based species delineation

**Fig. 6:**
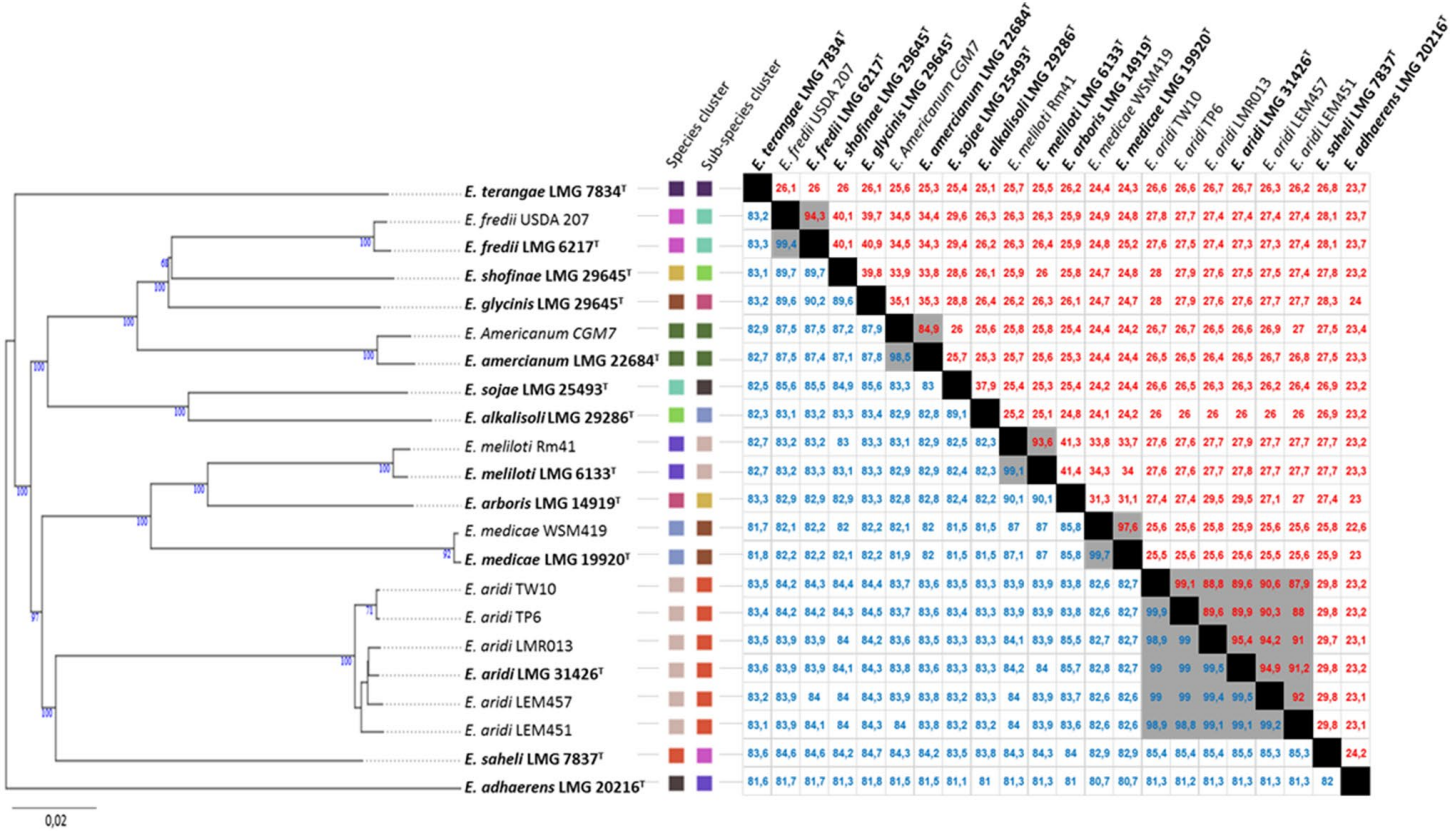
Genome-based species delineation. Phylogenomic tree inferred from GBDP distances obtained with TYGS. Branch lengths are scaled in terms of GBDP distance formula d5; numbers above branches are GBDP pseudo-bootstrap support values from 100 replications. Leaf labels are colored by affiliation to species and subspecies clusters. The matrix on the right shows ANI (lower left, blue font) and dDDH (upper right, red font) obtained upon pairwise comparisons. Values above thresholds for intraspecies comparisons are highlighted in gray. Type strains are indicated by bold font. Accession numbers of sequences used are reported in supplementary Table S3

Two genome-based species delineation methods were used to show that the species *Ensifer aridi* is distinct to already defined species. When we compared the genomes of the six *E. aridi* strains to available sequences of *Ensifer* type strains, both ANI and dDDH were well under the thresholds of 95% and 70%, respectively with  % values ranging from 81.2 to 85.7 for ANI and 23.1–29.8 for dDDH (Fig. 6). The phylogenomic tree obtained with TYGS automated pipeline showed, as expected, that the genomes of *Ensifer* strains belonging to the same species of *E. meliloti*, *E. medicae*, *E. fredii* or *E. americanum* presented ANI and dDDH above thresholds and TYGS classified them as distinct species accordingly. Finally, TYGS clustered all six *E. aridi* strains (sharing 98.8–99.9% ANI and 87.9–99.1 dDDH %) together within a yet not defined species whose closest relative is *E. saheli* (Fig. 6).

## Discussion

In this work, we characterized a collection of ten salt-tolerant rhizobial isolates originating from nodules of *Phaseolus filiformis* a wild bean native of northwestern Mexico growing on a sandy alkaline soil. These strains were also capable of developing the symbiosis with five cultivated bean varieties tested (Negro jamapa, peruano, pinto, vaquita and alubia).

Classification of rhizobia has gone through substantial changes in recent years due to the addition of new genera and species to this important group of bacteria. Rhizobia isolated from most *Phaseolus* species (including *P. filiformis*) generally belong to the genus *Bradyrhizobium* (Servín-Garcidueñas et al. 2014), except for *P. vulgaris*, where *Rhizobium* is found to be the most abundant symbiotic bacteria (Martínez et al. 1985; Piñero et al. 1988; Somasegaran et al. 1991; Souza et al. 1994; Silva et al. 1999; Parker 2002; Ormeño-Orrillo et al. 2006). Nevertheless, *Phaseolus vulgaris* is recognized as a non-selective host for nodulation (Michiels et al. 1998) and *Ensifer* strains or species (*E. fredii*, *E. medicae*, *E. meliloti* and *E. americanum*) have also been reported as *Phaseolus* symbionts in several continents (Bernal and Graham 2001; Mhamdi et al. 2002; Parker 2002; Wang et al. 2009; Mnasri et al. 2012; Adhikari et al. 2013; Verástegui-Valdés et al. 2014).

By analyzing the 16S rDNA sequences, we found that all the ten rhizobacteria isolated from *Phaseolus filiformis* belong to the genus *Ensifer*. 16S rDNA-based phylogenies indicated that eight of the isolates recovered here were, however, clustered together with the recently proposed species “*Ensifer aridi*” or *E. kostiense* which diverge from species already shown to develop an effective symbiosis with bean.

Taxonomical identifications based on 16S rDNA analysis is sometimes not powerful enough to discriminate the phylogenetic position of closely related strains or species due to high levels of sequence conservation (Acinas et al. 2004; Martens et al. 2007). The use of several loci present in single copies on genomes with greater sequence divergence and evenly distributed in the genome is an alternative approach that overcomes the drawbacks of 16S rDNA-based analyses (Vandamme et al. 1996; Stackebrandt et al. 2002; Zeigler 2003; Gevers et al. 2005; Thompson et al. 2005). To clarify the taxonomic position of *Phaseolus filiformis* isolates presented here, eight gene fragments (*atpD*, *dnaK*, *rpoB*, *glnA*, *gyrB*, *thrC*, *recA* and *glnII*) were sequenced and analyzed. The phylogenetic relationships obtained using *atpD*, *recA* and *glnII*, genes that have been sequenced in most *Ensifer* species showed that there might be several species among the isolated strains. One strain (LEM456) was grouped with *E. meliloti*, while the remaining strains isolated from *P. filiformis* were sharing a separate clade with strains of “*Ensifer aridi*” that, based on comparative genomics, includes the strains LEM451 and LEM457 (Le Queré et al. 2017). The use of six housekeeping genes (*atpD*, *rpoB*, *gyrB*, *thrC*, *recA* and *glnII*) for MLSA confirmed that the strain LEM456 is related to *Ensifer meliloti,* while the other strains clustered with “*Ensifer aridi”*; being different from already described *Ensifer* species nodulating *Phaseolus*, including *E. americanum*, a dominant *P. vulgaris* symbiont isolated from alkaline soils in Mexico (Verástegui-Valdés et al. 2014). Our results enlarge the spectrum of rhizobial species that can nodulate *Phaseolus* species and further support a shift toward *Ensifer* strains as preferred bean symbiont in alkaline soils.

Phylogenies obtained with symbiotic genes were not congruent with those obtained using housekeeping genes. According to the *nodC* phylogeny, all strains reported here clustered together including LEM456 which was, based on MLSA, clustering with *E. meliloti* strains. Surprisingly, the greatest *nodC* sequence similarities were found with *Ensifer* strains isolated from *Acacia macracantha* nodules in Peru which possess 16S rDNA sequences highly similar to the species *Ensifer fredii*, *E. americanum*, or *E. meliloti* (Cordero et al. 2016). However, the clade regrouping *nodC* sequences from *P. filiformis* and these *A. macracantha* isolates also showed some proximity with another cluster encompassing *nodC* sequences from *P. vulgaris* rhizobia belonging to the biovar *mediterranense* which included *E. americanum*, *E. fredii* and *E. meliloti* recovered from Tunisia, Spain or Mexico (Laguerre et al. 2001; Mnasri et al. 2007, 2012; Verástegui-Valdés et al. 2014). The *nifH*-based phylogenetic relationships of Mexican *P. filiformis* rhizobia indicated greater diversity, but they remained clustered together with sequences of *Ensifer* strains already shown to nodulate *Phaseolus* such as *E. americanum*. Interestingly the latter species was first described as *Acacia* symbionts in Mexico (Toledo et al. 2003), prior to being described as bean-nodulated rhizobia in Tunisian (Mnasri et al. 2007, 2012) and Mexican soils (Verástegui-Valdés et al. 2014) which demonstrates its broad geographical distribution and its capacity to exchange symbiotic gene arsenal to develop the symbiosis with multiple hosts. Based on the high similarity between *P. filiformis* rhizobia symbiotic genes sequences including that from the LEM456, our data support a local acquisition of this symbiotic gene pool through plasmid or genomic island transfers among Mexican bean-nodulating *Ensifer* strains and species which may even occur at the intergeneric level (Andrews et al. 2018; Verástegui-Valdés et al. 2014).

Within their natural habitat, rhizobial species are frequently subjected to detrimental effects such as water or salt-induced osmotic stresses or high temperatures which affect bacterial growth and root colonization. In the case of *E. americanum*, isolates tolerate 170 mM NaCl, but their growth is reduced at 260–340 mM and completely inhibited at 400 mM NaCl (Verástegui-Valdés et al. 2014). Higher salt tolerance levels were shown for *E. meliloti* bv. mediterranense strains which could grow in media containing salt concentrations greater than 750 mM (Mnasri et al. 2007). *E. meliloti* and *E. medicae* isolated from marginal soils affected by salt and drought, in arid regions of Morocco, have shown a large degree of variation for salt tolerance (Elboutahiri et al. 2010). *E. medicae* has shown good tolerance to NaCl (> 513 mM), compared to other rhizobia species (Zahran 1999). *E. meliloti* shows greater tolerance to salt (1711 mM NaCl) (Elboutahiri et al. 2010), indicating that saline soils naturally select strains more tolerant to salinity, and results in higher recovery of salinity-tolerant strains. All Mexican strains identified here were capable to grow at salt concentrations greater than 1.5% (about 250 mM), that was expected as they were recovered from beach saline sandy soil. Growth at salt concentrations greater than 400 mM was observed in few strains, and one of them, LEM451, was even capable of growing at 1000 mM NaCl; meanwhile, the most closely related *Ensifer* species *E. saheli* showed some growth up to 600 mM. Even though the tolerance to salt is highly variable both at the intra- or inter-specific levels, our results further demonstrate the relatively high salt tolerance of strains belonging to the genus *Ensifer*.

*Ensifer americanum*, *E. meliloti* bv. mediterranense and *E. saheli* strains have been shown to grow at pH ranging from 5.0 to 9.0, but not below 5.0 (Biswas et al. 2008; Verástegui-Valdés et al. 2014; this work). Despite some variations between strains studied in this report, the phenotypic characterization confirmed the particular adaptation of *Ensifer* toward alkaline pH; furthermore, as shown for other *Ensifer* species and strains, they appeared less adapted to acid pH. These data suggest that soil pH should be evaluated prior to application of rhizobial inocula to use the best adapted nitrogen-fixing bacteria in biofertilization programs.

The optimal growth of strains belonging to the genus *Ensifer* is generally obtained at temperatures around 25–33 °C (De Lajudie et al. 1994), but some species are capable of growing at temperatures ranges from 12 °C (Li et al. 2011) to 44 °C (De Lajudie et al. 1994). All *P. filiformis* strains presented here were tolerant to high temperatures (40 °C), similarly to *E. saheli* (De Lajudie et al. 1994) or to strains belonging to the proposed species *E. aridi* (Le Queré et al. 2017), to which most of the presented isolates were belonging. This capacity to withstand high temperatures was expected when considering the biome from which they were isolated.

*Phaseolus filiformis* strains were phenotypically characterized for their carbon source utilization. *Ensifer* strain LEM551 had a particular biochemical behavior. This strain was not capable of utilizing most carbon sources tested, being slightly divergent from the clade that grouped nine wild bean rhizobia by MLSA. That strain may represent a distinct species related to *E. aridi*. However, the most evolutionary distant strain LEM456 (related to *E. meliloti*) presented a similar profile to that of strains forming this dominant group which shows, based on the C sources tested, a lack of correlation between metabolic activity and taxonomy, and further support metabolic versatility among rhizobia as shown previously even at the intraspecies level in *Ensifer* (Biondi et al. 2009; De Lajudie et al. 1994).

## Conclusion

This study demonstrated that *P. filiformis*, a wild bean species native to the southwestern United States and northwestern Mexico is nodulated by rhizobial species belonging to the genus *Ensifer*. Among these wild bean rhizobia, one was related to *E. meliloti* and the others were clustering with “*Ensifer aridi”* a proposed new taxon yet only recovered from arid regions in America, Asia and Africa. Symbiotic gene-based phylogenies were incongruent with those obtained using housekeeping gene sequences suggesting a local exchange of this symbiotic gene through lateral gene transfers that mimics evolutionary histories of other *Ensifer americanum* and *E. meliloti* strains previously shown to nodulate *P. vulgaris*. Despite the high salinity conditions found in the beach sand where these symbiotic bacteria were isolated, salt tolerance was variable between isolates which nevertheless could all grow in presence of nearly 300 mM NaCl. These strains tolerate high pH rather than acidic conditions. They are also capable of growing at temperatures from 28 °C to at least 40 °C and were in majority capable of utilizing a wide range of carbohydrates and organic acids as sole carbon sources for growth. Capable of developing symbiosis with several *Phaseolus* species and cultivated bean varieties, these new strains present potential interest in biotechnology and should be further tested for their biofertilizing potential especially in alkaline soils subjected to salinization and heat, thanks to their phenotypic characteristics.

### Description of *Ensifer aridi* sp. nov.

*Ensifer aridi* (aridi, a diminutive of N.L. Fem *ariditas* is referring to the areas where all the strains from this new species where isolated from and which are subjected to droughts: these are the deserts of Thar in India, Merzouga in Morocco and here Baja California). Cells are Gram-negative, aerobic, motile and do not produce spores. Colonies are circular, from slightly to highly mucous, white opaque, convex and 1–3 mm diameter upon 48 h of growth on YMA medium at 30 °C. They can grow at particularly high temperatures (40 °C), tolerate pH ranging from 6 to 9.5 and all *Ensifer aridi* strains tested including the eight Mexican, the two Moroccan (LMR001, LMR013) and the two Indian (TP6, TW10) isolates were capable of growing using glycerol, l-arabinose, d-fructose, l-rhamnose, inositol, d-mannitol, d-maltose, d-sucrose, d-trehalose, d-raffinose, d-turanose or d-arabitol as sole carbon sources (Sakrouhi et al. 2016; Le Queré et al. 2017). The type strain is *Ensifer aridi* LMR001^T^ (= LMG 31426^T^; = HAMBI 3707^T^) isolated from Moroccan Merzouga desert sand dune (GPS coordinates: N 31°5′42.7″/W 3°57′56.1″) using *Vachellia gummifera* as a host (Sakrouhi et al. 2016). The genome sequence of the type strain is accessible under the Genbank accession LUAV00000000.1 together with those of five other strains including the Mexican strains LEM451 and LEM457 (Accessions LUFV00000000.1 and LUFW00000000.1, respectively) (Le Queré et al. 2017). Strains belonging to this new species present variable accessory genomes that explain the variable host ranges and C sources they can utilize (Le Queré et al. 2017). The DNA G + C content of strain LMR001^T^ was 61.7 mol%. Using the genome sequence of *E. aridi* LMR001^T^ as query, the highest ANI and dDDH values were found for the closest relative *E. saheli* LMG 7837^T^ with, respectively, 85.4% and 29.8% which clearly demonstrates that *Ensifer aridi* strains (NCBI:txid1708715) sharing more than 99% ANI and 87.9% dDDH belong to a new species within the genus *Enisfer*.

## Electronic supplementary material

Below is the link to the electronic supplementary material.

Supplementary material 1 (PDF 1069 kb)

Supplementary material 2 (PDF 1079 kb)

Supplementary material 3 (PDF 861 kb)

Supplementary material 4 (PDF 994 kb)
