# Supplementary material for: Diversity and phenotypic analyses of salt- and heat-tolerant wild bean Phaseolus filiformis rhizobia native of a sand beach in Baja California and description of Ensifer aridi sp. nov
Source: Arch Microbiol. 2019 Oct 28;202(2):309–22. doi: 10.1007/s00203-019-01744-7 (PMC7012998; doi:10.1007/s00203-019-01744-7)
Supplement: Supplementary file 1 — Supplementary material 1 (PDF 1069 kb) [file 203_2019_1744_MOESM1_ESM.pdf]

Archives of Microbiology

Diversity and phenotypic analyses of salt and heat tolerant wild bean *Phaseolus filiformis* rhizobia native of a sand beach in Baja California and description of *Ensifer aridi* sp. nov.

Guadalupe Rocha[1], Antoine Le Queré[2], Arturo Medina[1], Alma Cuéllar[1], José-Luis Contreras[3], Ricardo Carreño[1], Rocío Bustillos[1], Jesús Muñoz-Rojas[1], María del Carmen Villegas[4], Clémence Chaintreuil[2], Bernard Dreyfus[2], José-Antonio Munive[1#]

[1]Centro de Investigaciones en Ciencias Microbiológicas, Instituto de Ciencias, Benemérita Universidad Autónoma de Puebla. Av. San Claudio S/N, CP-72570, Puebla, México.

[2]IRD / CIRAD / UM2 / Supagro - UR 040 Laboratoire des Symbioses Tropicales et Méditerranéennes, F-34398 Montpellier, France

[3]Facultad de Arquitectura, Benemérita Universidad Autónoma de Puebla. Av. San Claudio S/N, CP-72570, Puebla, México.

[4]Helyx Affaires SC. Rumania 923-2. Col. Portales-Sur. Alcaldía Benito Juárez, CP-03300, Cd. de México, México.

[#]Corresponding author.

ORCID: https://orcid.org/0000-0003-4509-6563.

Mailing address: Centro de Investigaciones en Ciencias Microbiológicas, Instituto de Ciencias, Benemérita Universidad Autónoma de Puebla, Av. San Claudio S/N, CP72570, Puebla, México.

Phone: (+52-222) 2295500 – 2562. E-mail: joseantonio.munive@correo.buap.mx.

Keywords: Legume-rhizobium Symbiosis; *Ensifer*; wild bean; salt tolerance

Running title: Wild bean *Ensifer* native of hot arid environment in Baja California

Suppl. Table S1. Accessions of partial 16S rRNA and house keeping gene sequences used in the study. New sequences reported are indicated in bold and NU indicates that the correpsonding sequence was not used in the analysis. Type strains are indicated by a "T".

Strain	16S rRNA	*atpD*	*recA*	*glnII*	*gyrB*	*rpoB*	*thrC*	*dnaK*	*glnA*
Agrobacterium radiobacter LMG 140^T	AM181758	AM418785	AM182121	JN580718	NU	NU	NU	NU	NU
Bradyrhizobium elkanii LMG 6134^T	U35000.3	AM418752	AM182155	AY599117	NU	NU	NU	NU	NU
Bradyrhizobium japonicum LMG 6138^T	X66024	AM418753	AM182158	AF169582	NU	NU	NU	NU	NU
Ensifer adhaerens gv. C LMG 20216^T	AM181733	AM418746	AJ505595	HM997090	NU	NU	NU	NU	NU
Ensifer americanum LMG 22684^T	AB682471.1	AM418742	AM946572	GU994065	NU	NU	NU	NU	NU
Ensifer arboris LMG 14919^T	AM181744	AM418767	AM182130	HM997093	NU	NU	NU	NU	NU
Ensifer fredii LMG 6217 ^{T}	X67231	AM418761	AM182145	AF169591	NU	NU	NU	NU	NU
Ensifer garamanticus ORS 1400^T	AY500255	AM946546	AM946573	HM997091	NU	NU	NU	NU	NU
Ensifer kostiensis LMG 19227^T	AM181748	AM418771	AM182142	GU994063	AM418819	AM295369	AM181707	NU	NU
Ensifer kummerowiae CCBAU 71714^T	AY034028	GU994044	DQ411945	GU994062	NU	NU	NU	NU	NU
Ensifer medicae LMG 19920^T	L39882	AM418754	AM182135	AF169592	AM418802	AM295387	AM181700	NU	NU
Ensifer meliloti LMG 6133^T	X67222	AM418760	AM182133	DQ767676	NC_003047.1			NU	NU
Ensifer mexicanus HAMBI 2910^T	DQ411930	GU994045	DQ411951	GU994064	NU	NU	NU	NU	NU
Ensifer morelense LMG 21331^T	AM181737	AM418755	AJ505601	HM997095	NU	NU	NU	NU	NU
Ensifer numidicus ORS 1407^T	AY500254	AM946551	AM946576	HM997092	NU	NU	NU	NU	NU
Ensifer psoraleae CCBAU 65732^T	NR_133052	EU617988	EU622106	EU618006	NU	NU	NU	NU	NU
Ensifer saheli LMG 7837^T	X68390	AM418756	AM182138	AF169589	LNQB00000000			NU	NU
Ensifer sesbaniae CCBAU 65729^T	NR_133053.1	JX028795	JX028823	JX028809	NU	NU	NU	NU	NU
Ensifer sojae CCBAU 05684^T	GU593061	GU994042	GU994055	GU994060	NU	NU	NU	NU	NU
Ensifer terangae LMG 7834^T	X68388	AM418764	AM182153	AF169590	NU	NU	NU	NU	NU
Ensifer xinjiangensis LMG 17930^T	AM181732	AM418745	AM182148	HM997094	NU	NU	NU	NU	NU
Mesorhizobium mediterraneum LMG 17148^T	AM181745	AM418768	AM182157	AF169578	NU	NU	NU	NU	NU
Rhizobium giardinii R-4385^T	AM181755	AM418780	AM182123	EU488778	NU	NU	NU	NU	NU
Rhizobium leguminosarum LMG 14904^T	AM181757	AM418783	AM182125	JN580715	NU	NU	NU	NU	NU
Rhizobium rhizogenes LMG 150^T	AY945955	AM418786	AM182126	FJ816281	NU	NU	NU	NU	NU
Ensifer melilot i AK83	NU	FNYP00000000						NU	NU
Ensifer meliloti AC50a	NU	NJGD00000000						NU	NU
Ensifer meliloti bv. *mediterranense* 4H41	NU	AQWP00000000						NU	NU
Ensifer meliloti CCNWSX0020	NU	AGVV00000000						NU	NU
Ensifer meliloti CXM1-105	NU	PZMJ00000000						NU	NU
Ensifer sp. WSM1721	NU	AZUW01000000						NU	NU
Ensifer aridi PC2	NU	LATE01000000						NU	NU
Ensifer aridi LMR001	LUAV00000000								
Ensifer arid i LMR013	LUFU00000000								
Ensifer arid i JNVU TP6	LUFX00000000								
Ensifer aridi JNVU TW10	AZNX00000000								
Ensifer sp. LEM451	LUFV00000000								
Ensifer sp. LEM453	**KR780014**	**KR827451**	**KR873208**	**MK097261**	**KR873182**	**KR780004**	**KR873218**	**KR827569**	**KR873172**
Ensifer sp. LEM456	**KR780015**	**KR827452**	**KR873209**	**MK097262**	**KR873183**	**KR780006**	**KR873219**	**KR827570**	**KR873173**
Ensifer sp. LEM457	LUFW00000000								
Ensifer sp. LEM459	**KR780017**	**KR827454**	**KR873211**	**MK097264**	**KR873185**	**KR780008**	**KR873221**	**KR827572**	**KR873175**
Ensifer sp. LEM462	**KR780018**	**KR827455**	**KR873212**	**MK097265**	**KR873186**	**KR780009**	**KR873222**	**KR827573**	**KR873176**
Ensifer sp. LEM465	**KR780019**	**KR827456**	**KR873213**	**MK097266**	**KR873187**	**KR780010**	**KR873223**	**KR827574**	**KR873177**
Ensifer sp. LEM466	**KR780020**	**KR827457**	**KR873214**	**MK097267**	**KR873188**	**KR780011**	**KR873224**	**KR827575**	**KR873178**
Ensifer sp. LEM468	**KR780021**	**KR827458**	**KR873215**	**MK097268**	**KR873189**	**KR780012**	**KR873225**	**KR827576**	**KR873179**
Ensifer sp. LEM551	**KR780022**	**KR827459**	**KR873216**	**MK097269**	**KR873190**	**KR780005**	**KR873226**	**KR827577**	**KR873180**
