# Supplementary material for: Diversity and phenotypic analyses of salt- and heat-tolerant wild bean Phaseolus filiformis rhizobia native of a sand beach in Baja California and description of Ensifer aridi sp. nov
Source: Arch Microbiol. 2019 Oct 28;202(2):309–22. doi: 10.1007/s00203-019-01744-7 (PMC7012998; doi:10.1007/s00203-019-01744-7)
Supplement: Supplementary file 2 — Supplementary material 2 (PDF 1079 kb) [file 203_2019_1744_MOESM2_ESM.pdf]

Archives of Microbiology

Diversity and phenotypic analyses of salt and heat tolerant wild bean *Phaseolus filiformis* rhizobia native of a sand beach in Baja California and description of *Ensifer aridi* sp. nov.

Guadalupe Rocha[1], Antoine Le Queré[2], Arturo Medina[1], Alma Cuéllar[1], José-Luis Contreras[3], Ricardo Carreño[1], Rocío Bustillos[1], Jesús Muñoz-Rojas[1], María del Carmen Villegas[4], Clémence Chaintreuil[2], Bernard Dreyfus[2], José-Antonio Munive[1#]

[1]Centro de Investigaciones en Ciencias Microbiológicas, Instituto de Ciencias, Benemérita Universidad Autónoma de Puebla. Av. San Claudio S/N, CP-72570, Puebla, México.

[2]IRD / CIRAD / UM2 / Supagro - UR 040 Laboratoire des Symbioses Tropicales et Méditerranéennes, F-34398 Montpellier, France

[3]Facultad de Arquitectura, Benemérita Universidad Autónoma de Puebla. Av. San Claudio S/N, CP-72570, Puebla, México.

[4]Helyx Affaires SC. Rumania 923-2. Col. Portales-Sur. Alcaldía Benito Juárez, CP-03300, Cd. de México, México.

[#]Corresponding author.

ORCID: https://orcid.org/0000-0003-4509-6563.

Mailing address: Centro de Investigaciones en Ciencias Microbiológicas, Instituto de Ciencias, Benemérita Universidad Autónoma de Puebla, Av. San Claudio S/N, CP72570, Puebla, México.

Phone: (+52-222) 2295500 – 2562. E-mail: joseantonio.munive@correo.buap.mx.

Keywords: Legume-rhizobium Symbiosis; *Ensifer*; wild bean; salt tolerance

Running title: Wild bean *Ensifer* native of hot arid environment in Baja California

Suppl. Table S2. Accessions of partial symbiotic gene sequences used in the study. New sequences are indicated in bold and NU indicates that the correpsonding sequence was not used in the analysis.

Strain	host	Geographic origin	*nifH*	*nodC*
Ensifer adhaerens R7-601	*Jatropha* sp.	Singapore	KR075968	NU
Ensifer aridi LMR001	*Acacia gummifera*	Morocco	LUAV00000000	
Ensifer arid i LMR013	*Acacia tortilis*	Morocco	LUFU00000000	
Ensifer arid i JNVU TP6	*Tephrosia purpurea*	India	LUFX00000000	
Ensifer aridi JNVU TW10	*Tephrosia wallichii*	India	AZNX00000000	
***Ensifer* sp. LEM451**	***Phaseolus filiformis***	**Mexico**	LUFV00000000	
***Ensifer* sp. LEM453**	***Phaseolus filiformis***	**Mexico**	**KR873192**	**KR873200**
***Ensifer* sp. LEM456**	***Phaseolus filiformis***	**Mexico**	**KR873193**	**KR873201**
***Ensifer* sp. LEM457**	***Phaseolus filiformis***	**Mexico**	LUFW00000000	
***Ensifer* sp. LEM459**	***Phaseolus filiformis***	**Mexico**	**KR873195**	**KR873203**
***Ensifer* sp. LEM466**	***Phaseolus filiformis***	**Mexico**	**KR873196**	**KR873204**
***Ensifer* sp. LEM468**	***Phaseolus filiformis***	**Mexico**	**KR873197**	**KR873205**
***Ensifer* sp. LEM551**	***Phaseolus filiformis***	**Mexico**	**KR873198**	**KR873206**
Ensifer americanum 23C95	*Phaseolus vulgaris*	Tunisia	JN624732	NU
Ensifer americanum 23C40	*Phaseolus vulgaris*	Tunisia	JN624728	NU
Ensifer americanum 23C95	*Phaseolus vulgaris*	Tunisia	JN624732	NU
Ensifer americanum CCGM7	*Phaseolus vulgaris*	Mexico	NU	CP013053
Ensifer americanum CFNEI 73	*Acacia farnesiana*	Mexico	CP013109	CP013109
Ensifer fredii bv. mediterranense GR-06	*Phaseolus vulgaris*	Spain	NU	AF217269
Ensifer fredii NXT3	*Phaseolus vulgaris*	Mexico	NU	CP024309
Ensifer fredii GR64	*Phaseolus vulgaris*	Spain	JN034672	NU
Ensifer meliloti LMG 6133	*Medicago sativa*	USA	NC_003037	
Ensifer meliloti bv. *mediterranense* 4H41	*Phaseolus vulgaris*	Tunisia	DQ333890	DQ333891
Ensifer saheli LMG 7837	*Sesbania cannabina*	Senegal	LNQB01000082	
Ensifer terangae CB3126	*Leucaena leucocephala*	Mexico	NU	KJ128398
Ensifer sp. ATQ1	*Acacia macracantha*	Peru	KM192241	KM192232
Ensifer sp. ATQ3	*Acacia macracantha*	Peru	KM192243	NU
Ensifer sp. BR816	*Phaseolus vulgaris*	Brazil	NU	AJ518946
Ensifer sp. CEQ1	*Acacia macracantha*	Peru	KM192248	KM192239
Ensifer sp. CHU1	*Acacia macracantha*	Peru	KM192246	KM192237
Ensifer sp. CHU2	*Acacia macracantha*	Peru	KM192247	KM192238
Ensifer sp. FG01	*Phaseolus vulgaris*	Mexico	KJ921062	NU
Ensifer sp. GVPV04	*Phaseolus vulgaris*	Spain	NU	FJ462797
Ensifer sp. GVPV12	*Phaseolus vulgaris*	Spain	NU	FJ462796
Ensifer sp. LILM2009	*Phaseolus vulgaris*	Tunisia	FJ792815	NU
Ensifer sp. NG07A	*Phaseolus vulgaris*	Mexico	KJ921066	NU
Ensifer sp. SCAU191	*Leucaena leucocephala*	China	NU	JX073914
Ensifer sp. SCAU213	*Leucaena leucocephala*	China	NU	JX073921
Ensifer sp. SCAU224	*Leucaena leucocephala*	China	JQ362368	JX073925
Ensifer sp. SCAU228	*Leucaena leucocephala*	China	JQ362361	NU
Ensifer sp. STA1	*Acacia macracantha*	Peru	KM192244	KM192235
Ensifer sp. STA2	*Acacia macracantha*	Peru	KM192245	KM192236
Mesorhizobium alhagi CCNWXJ05 2	*Alhagi sparsifolia*	China	FJ481882	NU
Mesorhizobium amorphae CCNWGS0123	*Robinia pseudoacacia*	China	NU	JF907686
Mesorhizobium loti R46072	*Robinia pseudoacacia*	Belgium	FR850769	NU
Mesorhizobium robiniae CCNWYC132	*Robinia pseudoacacia*	China	EU849573	NU
Mesorhizobium robiniae CCNWYC147	*Robinia pseudoacacia*	China	EU849574	NU
Mesorhizobium robiniae CNWYC120	*Robinia pseudoacacia*	China	EU849572	NU
Mesorhizobium septentrionale CCBAU 11244	*Caragana microphylla*	China	GQ167293	NU
Mesorhizobium septentrionale W73	*Astragalus scaberrimus*	China	NU	JF730159
Mesorhizobium shangrilense CCBAU65321	*Caragana bicolor*	China	EU872234	NU
Mesorhizobium tarimense CCBAU83321	*Lotus tenuis*	China	EU252608	NU
Rhizobium acidisoli FH23	*Phaseolus vulgaris*	Mexico	KJ921065	NU
Rhizobium esperanzae CNPSo 661	*Phaseolus vulgaris*	Mexico	KY748058	NU
Rhizobium esperanzae CNPSo 668	*Phaseolus vulgaris*	Mexico	KY748056	NU
Rhizobium etli IE4771	*Phaseolus vulgaris*	Mexico	CP006988	NU
Rhizobium etli Mim1	*Mimosa affinis*	Mexico	JX863572	NU
Rhizobium etli RP330	*Phaseolus vulgaris*	Morocco	DQ413018	NU
Rhizobium etli CFN42	*Phaseolus vulgaris*	Mexico	NU	AF217268
Rhizobium etli RP218	*Phaseolus vulgaris*	Morocco	NU	DQ413007
Rhizobium etli RP346	*Phaseolus vulgaris*	Morocco	NU	DQ413008
Rhizobium gallicum RHM47	*Phaseolus vulgaris*	Morocco	NU	JQ085262
Rhizobium gallicum RP421	*Phaseolus vulgaris*	Morocco	NU	DQ413005
Rhizobium gallicum SPT1-23a	*Ammopiptanthus* sp.	China	KJ790195	NU
Rhizobium leguminosarum Gut 2	*Phaseolus vulgaris*	Nepal	AB740522	NU
Rhizobium leguminosarum Tsy 2	*Phaseolus vulgaris*	Nepal	AB740513	NU
Rhizobium leguminosarum Tsy 5	*Phaseolus vulgaris*	Nepal	AB740514	NU
Rhizobium leguminosarum LCS0306	*Phaseolus vulgaris*	Spain	NU	JF792203
Rhizobium leguminosarum LCS0313	*Phaseolus vulgaris*	Spain	NU	JF792202
Rhizobium leguminosarum LEV0613	*Phaseolus vulgaris*	Spain	NU	JF792204
Rhizobium lusitanum P2 2	*Phaseolus vulgaris*	Portugal	NU	HM852100
Rhizobium phaseoli ATCC14482	*Phaseolus vulgaris*	Spain	NU	HM441255
Rhizobium phaseoli VIAD8G	*Phaseolus vulgaris*	Dominican Republic	NU	KF638379
Rhizobium sophoriradicis CCBAU03470	*Sophora flavescens*	China	KM396547	NU
Rhizobium tarimense AS1-101a	*Ammopiptanthus* sp.	China	KJ790194	NU
Rhizobium undicola Ouran110	Rice root endophyte	India	JF738072	NU
Rhizobium vallis CCBAU65647	*Phaseolus vulgaris*	China	GU211767	NU
Rhizobium sp. SCAU203	*Leucaena leucocephala*	China	NU	KF613160
