# Supplementary material for: Diversity and phenotypic analyses of salt- and heat-tolerant wild bean Phaseolus filiformis rhizobia native of a sand beach in Baja California and description of Ensifer aridi sp. nov
Source: Arch Microbiol. 2019 Oct 28;202(2):309–22. doi: 10.1007/s00203-019-01744-7 (PMC7012998; doi:10.1007/s00203-019-01744-7)
Supplement: Supplementary file 3 — Supplementary material 3 (PDF 861 kb) [file 203_2019_1744_MOESM3_ESM.pdf]

Archives of Microbiology

Diversity and phenotypic analyses of salt and heat tolerant wild bean *Phaseolus filiformis* rhizobia native of a sand beach in Baja California and description of *Ensifer aridi* sp. nov.

Guadalupe Rocha[1], Antoine Le Queré[2], Arturo Medina[1], Alma Cuéllar[1], José-Luis Contreras[3], Ricardo Carreño[1], Rocío Bustillos[1], Jesús Muñoz-Rojas[1], María del Carmen Villegas[4], Clémence Chaintreuil[2], Bernard Dreyfus[2], José-Antonio Munive[1#]

[1]Centro de Investigaciones en Ciencias Microbiológicas, Instituto de Ciencias, Benemérita Universidad Autónoma de Puebla. Av. San Claudio S/N, CP-72570, Puebla, México.

[2]IRD / CIRAD / UM2 / Supagro - UR 040 Laboratoire des Symbioses Tropicales et Méditerranéennes, F-34398 Montpellier, France

[3]Facultad de Arquitectura, Benemérita Universidad Autónoma de Puebla. Av. San Claudio S/N, CP-72570, Puebla, México.

[4]Helyx Affaires SC. Rumania 923-2. Col. Portales-Sur. Alcaldía Benito Juárez, CP-03300, Cd. de México, México.

[#]Corresponding author.

ORCID: https://orcid.org/0000-0003-4509-6563.

Mailing address: Centro de Investigaciones en Ciencias Microbiológicas, Instituto de Ciencias, Benemérita Universidad Autónoma de Puebla, Av. San Claudio S/N, CP72570, Puebla, México.

Phone: (+52-222) 2295500 – 2562. E-mail: joseantonio.munive@correo.buap.mx.

Keywords: Legume-rhizobium Symbiosis; *Ensifer*; wild bean; salt tolerance

Running title: Wild bean *Ensifer* native of hot arid environment in Baja California

Supp. Table S3. Genome sequence accession, size and GC% of strains used for species delineation.

Strain	Base pairs	Percent G+C	Assembly accession
E. adhaerens LMG 20216T	7,280,736	62.3	GCA_000697965
E. alkalisoli LMG 29286T	5,973,017	62.2	GCF_001723275
E. americanum LMG 22684T	6,653,528	62.3	GCF_001651855
E. americanum CGM7	6,853,050	61.0	GCF_000705595
E. arboris LMG 14919T	6,849,525	62.0	GCA_000427465
E. aridi LMR001T (=LMG 31426T)	6,636,281	61.7	GCF_002078505
E. aridi LEM451	6,425,015	61.8	GCF_002093415
E. aridi LEM457	6,609,808	61.8	GCF_002093495
E. aridi LMR013	6,789,493	61.6	GCF_002093435
E. aridi TP6	6,848,280	61.6	GCF_002093525
E. aridi TW10	6,802,256	61.6	GCF_000510685
E. fredii LMG 6217 T	6,579,820	62.3	GCA_001461695
E. fredii USDA 207	6,962,831	62.2	GCF_000283895
E. glycinis LMG 29231T	6,039,283	62.4	GCF_001651865
E. medicae LMG 19920T	6,534,017	61.2	GCA_007827695
E. medicae WSM419	6,817,576	60.72	GCF_000017145
E. meliloti LMG 6133T	6,634,396	62.1	GCA_006539625
E. meliloti Rm41	7,414,029	62.1	GCF_000304415
E. saheli LMG 7837T	5,994,179	63.6	GCF_001651875
E. shofinae LMG 29645T	6,214,765	61.6	GCA_001704765
E. sojae LMG 25493T	5,964,336	62.0	GCF_000261485
E. terangae LMG 7834T (=USDA 4894T)	7,095,078	61.3	USDA4894v1*

Type strains are indicated by a "T"

* Assembly Accession retrieved from MaGe (https://www.genoscope.cns.fr/agc/microscope/home/index.php)
