# Supplementary material for: Diversity and phenotypic analyses of salt- and heat-tolerant wild bean Phaseolus filiformis rhizobia native of a sand beach in Baja California and description of Ensifer aridi sp. nov
Source: Arch Microbiol. 2019 Oct 28;202(2):309–22. doi: 10.1007/s00203-019-01744-7 (PMC7012998; doi:10.1007/s00203-019-01744-7)
Supplement: Supplementary file 4 — Supplementary material 4 (PDF 994 kb) [file 203_2019_1744_MOESM4_ESM.pdf]

Archives of Microbiology

Diversity and phenotypic analyses of salt and heat tolerant wild bean *Phaseolus filiformis* rhizobia native of a sand beach in Baja California and description of *Ensifer aridi* sp. nov.

Guadalupe Rocha[1], Antoine Le Queré[2], Arturo Medina[1], Alma Cuéllar[1], José-Luis Contreras[3], Ricardo Carreño[1], Rocío Bustillos[1], Jesús Muñoz-Rojas[1], María del Carmen Villegas[4], Clémence Chaintreuil[2], Bernard Dreyfus[2], José-Antonio Munive[1#]

[1]Centro de Investigaciones en Ciencias Microbiológicas, Instituto de Ciencias, Benemérita Universidad Autónoma de Puebla. Av. San Claudio S/N, CP-72570, Puebla, México.

[2]IRD / CIRAD / UM2 / Supagro - UR 040 Laboratoire des Symbioses Tropicales et Méditerranéennes, F-34398 Montpellier, France

[3]Facultad de Arquitectura, Benemérita Universidad Autónoma de Puebla. Av. San Claudio S/N, CP-72570, Puebla, México.

[4]Helyx Affaires SC. Rumania 923-2. Col. Portales-Sur. Alcaldía Benito Juárez, CP-03300, Cd. de México, México.

[#]Corresponding author.

ORCID: https://orcid.org/0000-0003-4509-6563.

Mailing address: Centro de Investigaciones en Ciencias Microbiológicas, Instituto de Ciencias, Benemérita Universidad Autónoma de Puebla, Av. San Claudio S/N, CP72570, Puebla, México.

Phone: (+52-222) 2295500 – 2562. E-mail: joseantonio.munive@correo.buap.mx.

Keywords: Legume-rhizobium Symbiosis; *Ensifer*; wild bean; salt tolerance

Running title: Wild bean *Ensifer* native of hot arid environment in Baja California

Supp. Table S4. Comparison of carbon utilization profiles of strains recovered from wild bean *P. filiformis* in Mexico.

Species	*E. aridi*														*E. terangae*	*E. saheli*	*E. meliloti*	*E. fredii*
**Strains**	**LEM451**	**LEM453**	**LEM457**	**LEM459**	**LEM462**	**LEM465**	**LEM466**	**LEM468**	**LMR001***	**LMR013***	**TP6***	**TW10***	**LEM551**	**LEM456**	**(n=20)****	**(n = 4) ****	**(n=3)****	**(n=2)****
**Carbon Sources**																		
glycerol****	+	+	+	+	+	+	+	+	+	+	+	+	-	+	+	+	+	+
erythritol	+	+	+	-	-	-	-	-	-	+	+	-	-	+	-	-	+	+/-
D-arabinose	-	+	+	+	+	+	+	+	ND	ND	ND	ND	-	+	+	+	+	+/-
L-arabinose****	+	+	+	+	+	+	+	+	+	+	+	+	+	+	+	+	+	+
ribose***	+	+	+	+	+	+	+	+	ND	ND	ND	ND	+	+	+	+	+	+
D-xylose***	+	+	+	+	+	+	+	+	ND	ND	ND	ND	-	+	+	+	+	+
L-xylose	-	+	+	+	-	-	-	+	ND	ND	ND	ND	-	-	+/-	+/-	+/-	-
adonitol	+	+	+	+	+	-	+	+	+	+	+	+	-	+	+	+	+	+
methyl-bD-xylanopiranoside***	+	+	+	+	+	+	+	+	ND	ND	ND	ND	+	+	ND	ND	ND	ND
D-galactose	+	+	+	+	+	+	+	-	+	+	+	+	-	+	+	+	+	+
D-glucose	+	+	+	-	+	-	+	-	+	+	+	+	-	+	+	+	+	+
D-fructose****	+	+	+	+	+	+	+	+	+	+	+	+	-	+	+	+	+	+
D-mannose	+	+	+	+	+	-	+	+	+	+	+	+	-	+	+	+	+	+
L-sorbose	-	+	+	+	-	+	+	+	ND	ND	ND	ND	-	-	-	-	+	-
L-rhamnose****	+	+	+	+	+	+	+	+	+	+	+	+	-	+	+	+	+	+
dulcitol	-	-	-	-	+	-	+	-	ND	ND	ND	ND	-	+	-	-	+	-
inositol****	+	+	+	+	+	+	+	+	+	+	+	+	+	+	+	+	+	+
D-mannitol****	+	+	+	+	+	+	+	+	+	+	+	+	+	+	+	+	+	+/-
D-sorbitol	+	+	+	+	+	+	-	+	+	+	+	+	-	+	+	+	+	+/-
methyl-aD-mannopyranoside	-	-	-	-	-	-	+	-	ND	ND	ND	ND	-	-	ND	ND	ND	ND
methyl-aD-glucopyranoside	-	+	+	-	-	-	+	+	ND	ND	ND	ND	-	+	ND	ND	ND	ND
N-acetylglucosamine	+	-	+	-	-	-	-	+	+	+	+	+	-	-	+	+	+	+
amygdalin	-	-	-	-	-	-	+	-	ND	ND	ND	ND	-	-	+/-	-	-	-
arbutin	-	+	+	+	+	+	+	+	ND	ND	ND	ND	-	-	+	+	+	+
esculin ferric citrate	-	+	+	+	+	+	+	+	ND	ND	ND	ND	-	+	-	-	-	-
salicin***	+	+	+	+	+	+	+	+	ND	ND	ND	ND	+	-	+/-	+/-	+	+
D-cellobiose	+	+	+	+	+	-	+	+	+	+	+	+	-	+	+	+	+	+
D-maltose****	+	+	+	+	+	+	+	+	+	+	+	+	+	+	+	+	+	+
D-lactose	+	+	+	+	-	-	+	+	+	+	+	+	+	+	+	+	+	+
D-melibiose	+	-	+	-	+	-	+	+	+	+	+	+	-	+	+	+	+	+
D-sucrose****	+	+	+	+	+	+	+	+	+	+	+	+	-	+	+	+	+	+
D-trehalose****	+	+	+	+	+	+	+	+	+	+	+	+	-	-	+	+	+	+
inulin	-	-	-	-	-	+	+	-	ND	ND	ND	ND	-	+	-	-	-	-
D-melezitose	-	-	+	-	-	-	-	-	ND	ND	ND	ND	-	+	+/-	+/-	+	+/-
D-raffinose****	+	+	+	+	+	+	+	+	+	+	+	+	-	+	+	+	+	+
starch	-	-	-	-	-	-	+	-	ND	ND	ND	ND	-	+	-	-	-	-
glycogen	-	-	-	-	-	-	-	-	-	-	-	-	-	-	-	-	-	-
xylitol	-	+	-	+	+	+	+	+	-	-	-	-	-	-	+	-	+	-
gentiobiose	+	-	+	+	-	-	+	+	+	+	+	+	-	+	+/-	+/-	+	+
D-turanose****	+	+	+	+	+	+	+	+	+	+	+	+	-	+	+	+	+	+
D-lyxose***	+	+	+	+	+	+	+	+	ND	ND	ND	ND	+	+	+/-	+/-	+	-
D-tagatose	-	-	-	+	+	-	+	+	ND	ND	ND	ND	-	-	-	-	+	-
D-fucose***	+	+	+	+	+	+	+	+	-	+	+	+	-	+	-	-	+/-	+/-
L-fucose***	+	+	+	+	+	+	+	+	ND	ND	ND	ND	-	+	ND	ND	ND	ND
D-arabitol****	+	+	+	+	+	+	+	+	+	+	+	+	-	+	+	+	+	+
L-arabitol	-	+	+	+	+	+	+	+	ND	ND	ND	ND	-	+	-	-	+	+
potassium gluconate	+	+	-	-	-	+	+	-	ND	ND	ND	ND	-	-	+/-	+/-	-	-
potassium 2-ketogluconate	+	-	-	-	-	-	+	-	ND	ND	ND	ND	-	-	+	+/-	+	-
potassium 5-ketogluconate***	+	+	+	+	+	+	+	+	ND	ND	ND	ND	+	+	-	-	-	-

+ indicates Carbon utilization by the strain(s)

- indicates that the strain(s) are not using the corresponding C source

+/- indicates contrasted C source utilization from strains belonging to the described species tested in De Lajudie et al., (1994)

ND: not determined

* Data retrieved from Le Quéré *et al.*, (2017)

** Data retrieved from De Lajudie *et al.*, (1994). Numbers in parentheses indicate the number of strains studied

*** indicates carbon sources that can be utilized by the 8 *Ensifer aridi* strains isolated from *Phaseolus filiformis*

**** indicates carbon sources that can be utilized by all *Ensifer aridi* isolates tested (8 Mexican, 2 Moroccan and 2 Indian isolates)
